# A Vision-Based Counting and Recognition System for Flying Insects in Intelligent Agriculture

**DOI:** 10.3390/s18051489

**Published:** 2018-05-09

**Authors:** Yuanhong Zhong, Junyuan Gao, Qilun Lei, Yao Zhou

**Affiliations:** College of Communication of Engineering, Chongqing University, Chongqing 400044, China; 20144134@cqu.edu.cn (J.G.); iLot9s0@163.com (Q.L.); zhouyao@cqu.edu.cn (Y.Z.)

**Keywords:** flying insect, counting and recognition system, YOLO, SVM, Raspberry PI

## Abstract

Rapid and accurate counting and recognition of flying insects are of great importance, especially for pest control. Traditional manual identification and counting of flying insects is labor intensive and inefficient. In this study, a vision-based counting and classification system for flying insects is designed and implemented. The system is constructed as follows: firstly, a yellow sticky trap is installed in the surveillance area to trap flying insects and a camera is set up to collect real-time images. Then the detection and coarse counting method based on You Only Look Once (YOLO) object detection, the classification method and fine counting based on Support Vector Machines (SVM) using global features are designed. Finally, the insect counting and recognition system is implemented on Raspberry PI. Six species of flying insects including bee, fly, mosquito, moth, chafer and fruit fly are selected to assess the effectiveness of the system. Compared with the conventional methods, the test results show promising performance. The average counting accuracy is 92.50% and average classifying accuracy is 90.18% on Raspberry PI. The proposed system is easy-to-use and provides efficient and accurate recognition data, therefore, it can be used for intelligent agriculture applications.

## 1. Introduction

Agriculture plays an important role in economic growth and the improvement of crop yield is of great significance [1]. However, insect pests can affect the metabolic processes of crops to degrade crop yield and quality, which may further hinder the development of agriculture [2]. In order to ensure high crop yields, agricultural workers tend to use pesticides according to a schedule rather than the likelihood of pests’ presence in the wild [3]. Thus, this not only causes a large number of pesticide residues in agricultural commodities, but also brings great pressure to the ecological environment [4]. The over-use of pesticides is partly because information about pest species and densities cannot be provided in a timely and accurate way. In contrast, if the information is provided in a timely fashion, it could be possible to take proper prevention steps and adopt suitable pest management strategies including the rational use of pesticides [5,6]. Therefore, information about pest species and densities is very important and necessary.

Traditionally, the information about pest species and densities is acquired mainly through the visual judgment of humans [5]. In this way, workers compare a pest’s shape, color, texture and other characteristics with the information recorded by many experts. However, the pest record is usually either inaccurate or imperfect, and some degrees of pest damage are difficult to describe in words. Consequently, manual counting is typically time consuming, labor intensive and error-prone [5,7] and therefore, it is urgent and significant to establish an automatic, efficient and accurate pest identification system. Fortunately, due to the rapid development of digital image technology, there is a growing tendency of utilizing machine vision technology to solve these problems with promising performance in the agriculture research field [5].

To evaluate pest species and densities based on machine vision, the first thing to do is to get a clear image of the insect. However, insects are always moving, and it is hard to get clear images directly, especially for flying insects. Some studies [8,9,10,11] chose insect specimens which were well preserved in an ideal laboratory environment to avoid the problem, and images can be captured at high resolution, but since less environmental issues are considered in this method, it is limited in specific applications. A more practical method is to use traps, in this way, insects can be attracted by a variety of ways based on light [12], color [5,13], pheromones [7,14], etc. Sticky traps [15] are usually used to stick insects and fix them, and then, the sticky trap is photographed. In [5,12,13,14,15,16] researchers collected insects in the wild with traps and acquired images in an ideal lab environment by hand. This method can this get clear images since the shooting settings, such as illumination and angle, can be adjusted according to the requirements, however, shooting in a manual way was still time consuming and labor intensive. Only a small number of studies, such as [7], collected insects with trap and acquired images in the wild to train a counting and identification model, which can enhance the applicability in the wild.

Based on the images of sticky traps, image segmentation was then employed before recognition. Image segmentation is the process of partitioning an image into multiple segments to extract the region(s) of interest. Cho et al. [5] collected thrips, whiteflies and aphids in a greenhouse through a yellow sticky trap, and separated the pests from the background by the iterative method. In [13], Sun collected whiteflies and thrips using yellow and blue sticky traps, then segmentation was performed by choosing an appropriate color filter. Vakilian et al. [17] extracted insect pests from the background through a canny edge detection segmentation process. The segmentation and detection results can be taken as the rough counting results of insects with an additional algorithm, such as connected components labeling [5], however, the accuracy is easily affected by illumination, impurities, and so on.

Effective recognition requires appropriate features. Usually, the global features, such as color, morphology and texture, and local features were extracted for identification. Cho et al. [5] used size and color components to identify whiteflies, aphids and thrips. Gassoumi et al. [18] extracted the morphology features, including compactness, aspect ratio, extent, etc., to classify 12 kinds of common cotton pests. Vakilian et al. [17] extracted four morphological features and three texture features of the pests, and successfully completed the identification of beet armyworms. Different recognition methods could be utilized based on the extracted features including, SVM [9,19], artificial neural network (ANN) [11,17,18,20], k-nearest neighbors (KNN) [21], etc. In [20], a method based on ANN was used for identification of butterfly species. Larios et al. [19] proposed an image classification method based on extracting image features using Haar random forests and combining them with a spatial matching kernel SVM to identify stonefly species. In recent years, feature extraction methods have gradually shifted from manual design to data-driven methods based on deep learning. In [7], a convolutional neural network (CNN) structure is designed to extract features of moth and identify it, however, deep learning requires lots of samples to get satisfied performance.

Although some relevant studies have made great progress, their research still largely remains in a theoretical phase with less attention to actual application scenarios because of two main challenges: firstly, non-deep learning methods are popularly adopted to count and identify insects, but the accuracy is sometimes limited [5,9]. Now, deep learning methods are gradually applied to this task, however, the requirement for training data amount is pretty high [7], which may be hard to satisfy for some species of insects. Hence, the generalization ability for different species of insects is limited. Secondly, most of current researches use insect images collected in an ideal lab environment without implementation in the wild [5,8,9,10,18,19,22]. Although a small number of studies [7] use insect images collected in the wild, these images at high resolution must be transmitted to a server where the counting and identification task is completed. Moreover, none of these identification results have been combined with other natural information for the convenience of providing early warnings. Therefore, even though the test results can be promising, the system is limited to specific applications.

In this study we designed a unique automatic pest counting and identification system to try to eliminate the above problems. Firstly, a yellow sticky trap is installed in the wild to trap flying insects and a digital camera is set up to collect images. Secondly, we take flying insects as one class, and YOLO object detection based on deep learning is adopted to make detection and coarse counting of flying insects. A SVM classifier is then used to perform classification and fine counting using the detection results. Based on the combination of YOLO deep learning method and SVM classifier, the requirement for training data is minimized. Then, this system is implemented on a Raspberry PI system, and the test results can be sent to an agricultural monitoring service platform, which is the basis of providing precise prevention and treatment methods based on the combination of pest information and other environmental information. Based on this edge computing design, the computation pressure on the server is alleviated and the network burden is largely reduced.

The reminder of the paper is organized as follows: Section 2 describes the overall design of the proposed system. Section 3 mainly studies the detection and coarse counting algorithm of flying insects based on the YOLO network. In Section 4, the classification and fine counting method of SVM is studied. Section 5 explains the test results based on the different methods. Finally, Section 6 gives the conclusions.

## 2. System Overview

In this section, the hardware and software frameworks of the counting and recognition system are studied. The counting and classification result of the proposed system can be sent to a meteorological station, which is the data acquisition equipment for agricultural monitoring service platforms. This service platform is also introduced for the integrality of the system.

### 2.1. Agricultural Monitoring Service Platform

**Figure 1:**
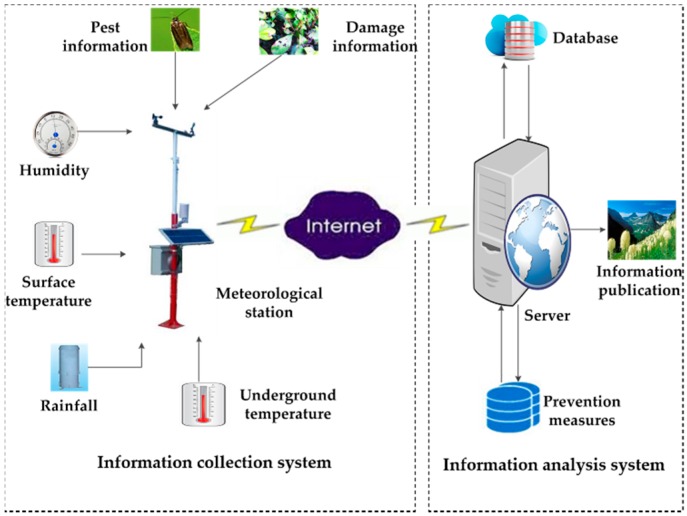
The components of agricultural monitoring service platform.

The counting and recognition system of flying insects which can help monitor the population dynamics of pests is a subsystem of an information collection platform for agricultural monitoring services. The agricultural monitoring service platform is an integrated platform that utilizes sensor technology, communication technology, cloud technology, big data technology, etc. This platform includes pest monitoring sites, weather stations, sensor base stations, etc., which have been established to realize the monitoring, analysis and comprehensive prevention of crops in the wild. The component of the service platform is shown in Figure 1.

**Figure 2:**
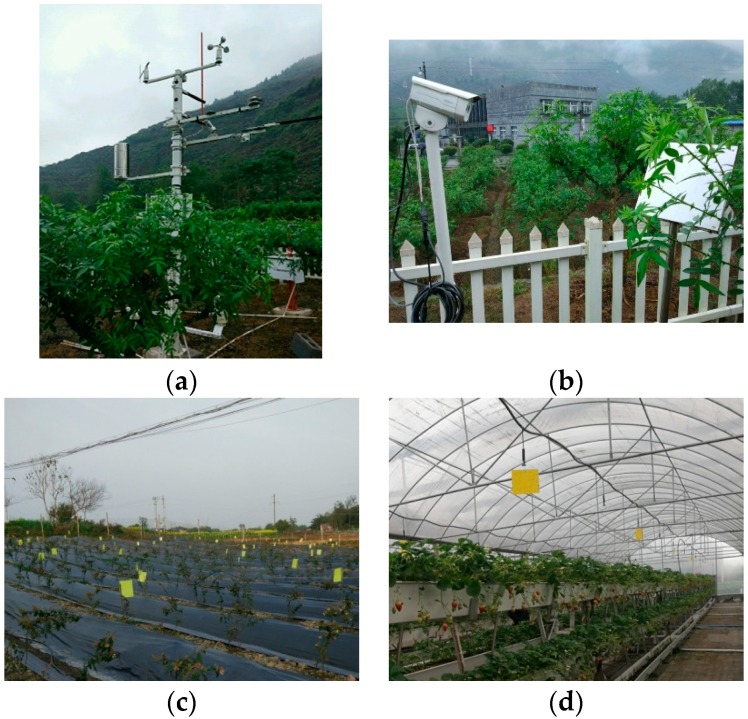
Illustration of meteorological station and yellow sticky trap (**a**) Meteorological station; (**b**) Equipment for trapping insects and collecting images; (**c**) Flower planting base; (**d**) Strawberry greenhouse.

The main functions of the service platform are data acquisition, data analysis and information publish. The information collection system collects the environment information and pest information through a variety of sensors and control equipment. The environment information includes light, temperature, surface temperature, underground temperature, rainfall, soil moisture, etc. The pest information includes pest species, crops species, damage degree, etc. An instance of information collection station is shown in Figure 2a. Based on the pest data and other natural data, maybe several years-worth, the big data technology can build a relationship of the pests with environmental factors, then the information analysis system can make a prediction of pest population dynamics according to the data, and precise prevention and treatment methods could also be provided. Eventually, the results can be published to users and management departments.

### 2.2. Hardware Settings 

The counting and recognition of flying insect subsystem is an important component of the service platform. Our hardware system is mainly composed of image acquisition and an image processor. The function of the image acquisition system is to trap flying insects and collect images. The image processor is then used to complete the task of flying insect counting and classifying. Then, recognition results are sent to the meteorological station in Figure 2a via a GPRS model.

In order to capture clear images, a metal board used to paste a yellow sticky trap is installed and a camera is set up in front of it at a proper distance as shown in Figure 2b. All of our images are collected from a strawberry greenhouse and flower planting base as shown in Figure 2c,d.

We adopt Raspberry Pi 2 Model B as the image processor, which has sufficient computing capability and external expansion interfaces, and adopt the Raspberry Pi Camera Module v2 as the camera. The v2 Camera Module has a Sony IMX219 8-megapixel sensor, which can be used to take high-definition video, as well as still photographs. The photos are taken in natural light during the day, so there is no need for an external light source or internal flashlight.

### 2.3. Software Framework

**Figure 3:**
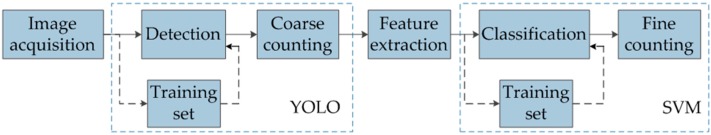
Flow chart of counting and recognition system of flying insects.

The software flow chart of the counting and recognition system is shown in Figure 3. The main process is composed of image acquisition, training set building, flying insect detection, coarse counting of flying insects, feature extraction, classification and fine counting of classified flying insects.

• Image acquisition

Obtaining a clear digital image of flying insect is the premise for counting and recognition, so the trapping device and the image acquisition device should be suitable for different species of flying insects and different field environments.

• Detection

The purpose of this module is to detect flying insects. Since image acquisition process is easily affected by illumination, camera out of focus, impurities and other factors [7,23], the detection algorithm needs to have strong anti-interference ability and reliability. Although deep learning method such as YOLO can usually obtain good performance, it is difficult to get enough samples of some specific insects. Therefore, we propose the resolution of regarding all species of flying insects as one class, using deep learning method to detect and coarsely count flying insects, and providing detecting results to SVM to make fine classification. In this way, the problem of insufficient samples is solved. Besides, the system is easy to add or change identified categories of flying insects without re-training the whole system.

• Training set

In this study, the insects over 10,000 are manually labeled using rectangle as the dataset for YOLO. And 30 × 30 images are collected as the training set for SVM. Both positive samples and negative samples are included in the dataset. And the samples are validated by multiple experts.

• Coarse counting

In this process, the number of flying insects can be obtained, but the specific species of flying insect is not provided.

• Feature extraction

Feature extraction is concerned with mathematical tools for quantitatively describing an object [18]. In order to obtain overall feature information, multiple features are chosen in this work to establish the feature space.

• Classification

Based on the extracted features, we use SVM to classify the detection results of YOLO into 7 classes including bees, flies, mosquitoes, moths, chafers, fruit flies and other flying insects.

• Fine counting

In order to quantitatively describe the intensity of different species of flying insects and to accurately monitor their population dynamics, it is necessary to obtain the number of every species of flying insects.

## 3. Detection and Coarse Counting of Flying Insects Based on YOLO

Since the yellow sticky trap is installed in the wild, the collected images can be easily influenced by variations in illumination and many impurities such as insect excrement, dead leaves, water droplets and mud spots [7,23]. Hence, any counting algorithm needs to adapt to this complex environment. Thus it can be realized in traditional ways, such as connected components labeling algorithms [5], or more advanced, deep learning algorithms [7]. In this section, the detection and coarse counting method of flying insects based on the YOLO deep learning method is studied, and its performance is compared with the traditional method in Section 5.1.

### 3.1. YOLO Network Architecture

YOLO [24] is an end-to-end convolution neural network commonly used in object detection and recognition. The main features of YOLO include high speed and accuracy. YOLO uses a single convolution neural network to simultaneously predict the bounding boxes and class probabilities for these boxes directly from the entire image. Therefore, compared with other detection and identification methods in which objects detection is divided into region prediction, class prediction and other processes, YOLO’s region prediction and class prediction are integrated in a network to improve the detection speed [24].

**Figure 4:**
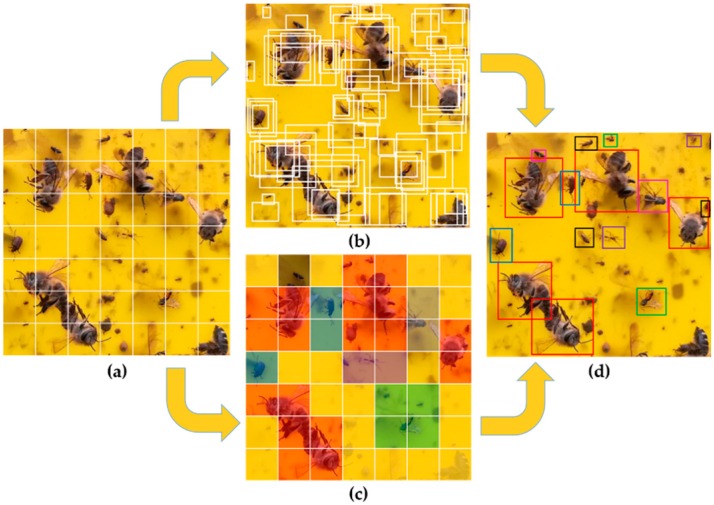
Detection model of YOLO (**a**) Divided in to cells with equal size; (**b**) The predicted bounding boxes of cell; (**c**) Class probability map; (**d**) Final detection of YOLO.

Detection is regarded as a regression problem in YOLO, and the process of object detection includes about three steps. Firstly, the input image is divided into a S × S grid as shown in Figure 4a. Then, each grid predicts *B* bounding boxes of objects if the object’s center falls into the grid as shown in Figure 4b.

Each bounding box includes five parameters: the center point coordinates (x,y) relative to the bounds of the grid cell, width and height (w,h) relative to the whole image and confidence. And the confidence is the product of the probability that the bounding box contains the object of interest *Pr(Object)* and Intersection Over Union (IOU):

(1) $$confidence=Pr(Object)\times IOU_{pred}^{truth}$$

$IOU_{pred}^{truth}$ refers to the intersection of predicted box and ground truth box. If there is no object in that grid cell, confidence=0. Otherwise, confidence is expected to be equal to IOU. Supposing an object exists in a grid cell, the grid cell also predicts C conditional class probabilities $Pr(Class_{i}|Object),\;i=1,\;2,\;\cdots ,\;C$. C conditional class probabilities are predicted only once in a grid cell, which means B bounding boxes share the same conditional class probability. And the Class probability map is shown in Figure 4c. As a result, the final output of YOLO is a S × S × (B × 5 + C) tensor. Figure 4d shows the final output of the YOLO network. Thirdly, the threshold is set to remove bounding boxes with relatively low class-specific confidence score, and non-maximum suppression (NMS) is used to remove the redundant bounding boxes. The class-specific confidence score can be obtained by the product of conditional class probability and the individual box confidence. This class-specific confidence score reflects how possible the object belonging to the class exits in the box and how suitable the predicted box is for the object:

(2) $$class\_confidence=Pr(Class_{i}\;|Object)\times confidence$$

**Figure 5:**
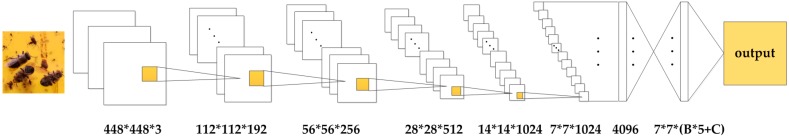
Architecture of YOLO.

The YOLO network is based on the convolution neural network structure. The structure of YOLO includes 24 convolutional layers and two fully connected layers. Convolutional layers are used to extract image features and fully connected layers are used to predict the output probability [24]. There are two kinds of convolution kernels in the convolutional layer which are 3 × 3 and 1 × 1. The architecture of YOLO is shown in Figure 5.

The resolution of input image is 448 × 448. S is set to be 7. The parameter B of the output tensor is 2, which means each grid predicts two bounding boxes of objects. As for C, in the detection and coarse counting process, it is set to be 1, which means YOLO is only used to detect if the object is flying insect or not, without any specific classification of flying insects.

### 3.2. YOLO Network Training

In order to speed up the network training process, the pre-training model of original YOLO system is adopted in this work. Based on the 1000 classes of ImageNet dataset, 20 convolutional layers, one pooling layer and one fully connected layer are trained for about 200,000 times. Hence, most of the parameters in the network are adjusted to an acceptable range. Then, new convolution layer is added based on the pre-training model to convert the classification model into detection model, and the network parameters are fine-tuned according to the flying insect dataset.

**Figure 6:**
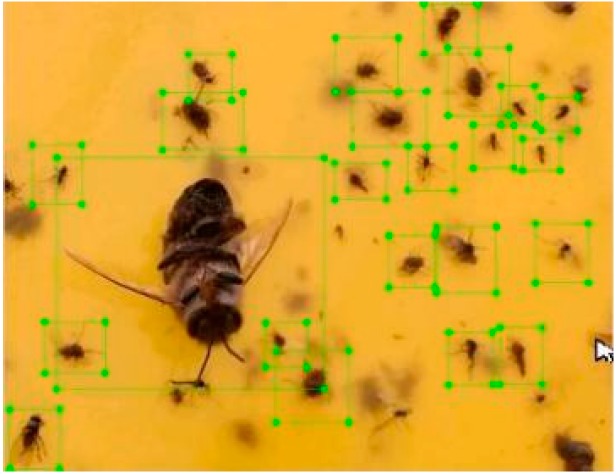
Labeling of flying insects.

As for the training dataset of flying insect, since the original image with a resolution of 3280 × 2464 collected by the camera is too large to be the input of the network, the original image is cropped and scaled to a size of 448 × 448 in order to reduce the network computing amount. In this process, we needn’t to make sure the numbers of every species of flying insects are close to each other, on the contrary, we regard flying insects as one class to make coarse detection and counting. The training dataset is up to 3000 images, which are manually labeled with the LabelImg tool. The labeling of flying insects is presented in Figure 6. Besides, in order to avoid overfitting that may occur in deep network training, we use rotation, scaling, flip, translation and other transformations as well as contrast adjustment, noise addition, and other operations to expand the training dataset to 12,000. Then we random select 10,000 images from 12,000. Consequently, the number of labeled images is 10,000 including 8000 training dataset, 1000 validation dataset and 1000 testing dataset.

In our work, stochastic gradient descent (SGD) method is used, and the network is trained for 50,000 iterations. Batch size is set to be 8, momentum and weight decay are set to be 0.9 and 0.000001, respectively. The initial learning rate is set to be 0.001, and the coefficient is multiplied by 0.1 after every 10,000 iterations.

## 4. Classification and Fine Counting of Flying Insects Based on SVM

There are various classification methods which are widely used in classification of flying insects such as SVM [9,19], ANN [11,17,18,20], CNN [7], etc. Simply, it can be generalized as two categories: deep learning method and non-deep learning method. Considering the high requirement for training dataset of deep learning method, in this section, SVM are designed to classify six species of flying insects, and its performance is compared with YOLO in Section 5.1.

### 4.1. Feature Extraction

**Figure 7:**
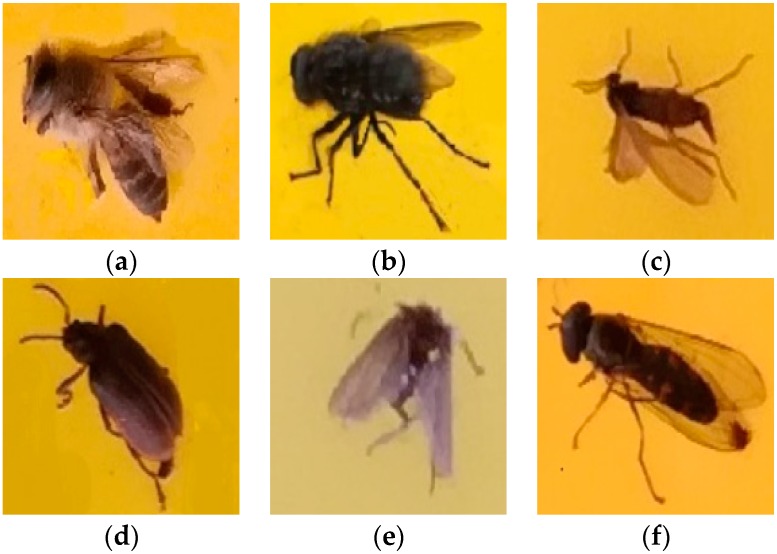
Flying insect images (**a**) Bee; (**b**) Fly; (**c**) Mosquito; (**d**) Chafer; (**e**) Moth; (**f**) Fruit fly.

In this work, six species of flying insects, including bees, flies, mosquitoes, chafers, moths and fruit flies, are selected to train and test the SVM classifier and they are presented in Figure 7.

Feature extraction is necessary for SVM to find key features that can reflect the species of flying insects showed in Figure 7. So the following points should be considered. Firstly, the extracted features should have intra-class similarity and inter-class diversity; secondly, since flying insects in different species have some similarities in a certain feature, it is import to extract a variety of features [18]; thirdly, the features should be easily acquired and represent taxonomic information [9]. Three kinds of global features including shape, texture and color feature and one local feature are considered in this work:

• Shape Feature Extraction

Shape features includes geometric features and Hu invariant moments [12,25]. Due to the large difference in area and circumference of the same species of flying insect, four geometrical features are chosen in this paper including complexity, duty cycle, eccentricity and extension rate.

• Texture Feature Extraction

Texture feature is a regional feature, which reflects greyscale statistics, surface structure change information and spatial distribution information of the image [26,27]. To extract texture features, gray level L is set to be 64 and scan angle is set to be 0°, 45°, 90° and 135°. The grey-level co-occurrence matrix (GLCM) [28] of R, G and B color components is calculated, respectively. Then, texture parameters are deduced including contrast, correlation, second order moment and entropy. At the same time, their mean and variance are calculated.

• Color Feature Extraction

Color is important because different kinds of flying insects and different parts of them are in different colors, and it is invariable to position, rotation and scale. Commonly used color feature [29] extraction methods include color histogram [30], color moments [31], color correlogram [32], etc. In this work, R, G, B color components are extracted and each component has three lower order moments, so nine color moments are extracted from one target.

• HOG Feature Extraction

HOG was proposed by Dalald et al. [33]. It is insensitive to changes in illumination and scale, which makes it widely used in many fields. To extract HOG features, the resolution of image, cell and block is set to be 64 × 128, 32 × 32 and 64 × 64 respectively in this work. The shape of block is R-HOG and each block is made up of four cells. Nine bin histograms are used to calculate the gradient of each cell. Block overlap rate is set to be 50% and the dimension of HOG features is 108.

### 4.2. Construction of the SVM Model

**Figure 8:**
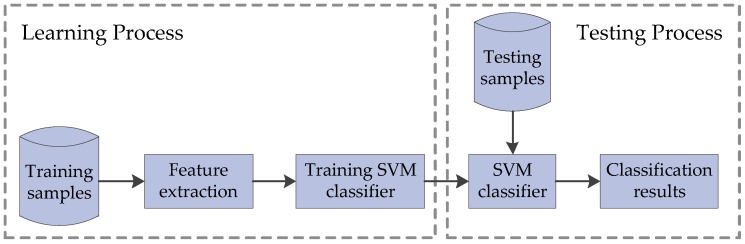
Training and testing process of SVM.

SVM [34], which was proposed by Cortes et al. in 1995, is a supervised machine learning method used for classification. SVM can solve the problems of small sample, high dimensionality, non-linearity and so on. The training and testing process of SVM is shown in Figure 8.

**Figure 9:**
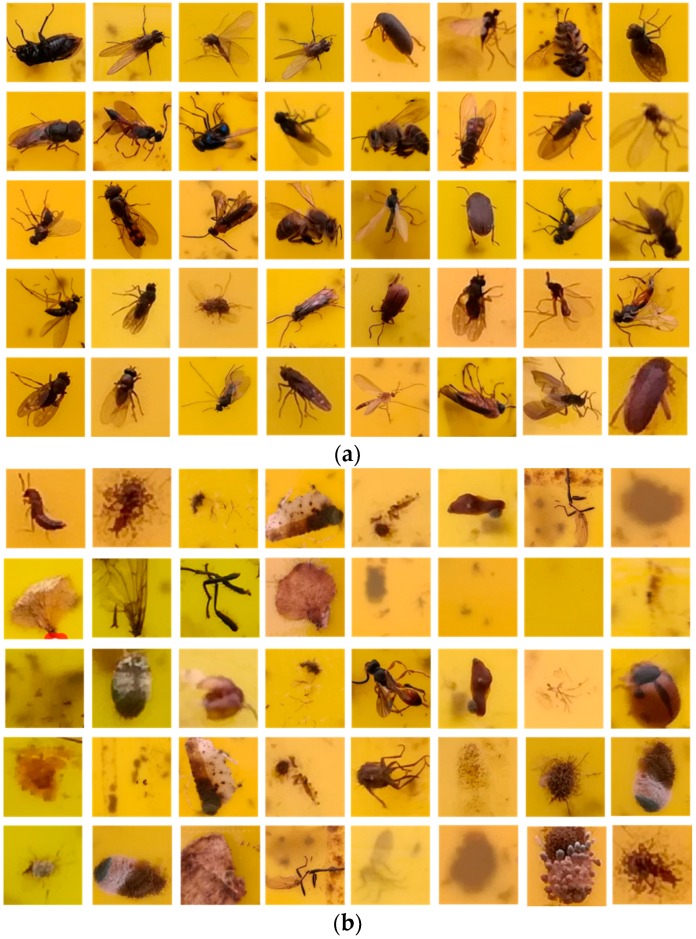
Positive and negative examples (**a**) Positive examples; (**b**) Negative examples.

For the training dataset of SVM, the number of flying insects in different species should be roughly equivalent. What’s more, flying insects of the same species should have different gestures. However, in some seasons, some flying insects do not appear widely, and it is time consuming to collect different species of flying insects. Considering above factors, the data set size of six species of flying insects is set to be 700. Since the size is limited, we perform data augmentation [7] to increase the number to be 7000 which is used as positive examples. The positive examples are then divided into a training set including 4900 samples and a testing set including 2100 samples. One thousand randomly selected images are increased to 10,000 by data augmentation which is used as negative examples. Both positive and negative examples employing 30 × 30 pixels images are used to train the SVM classifier. And some positive and negative examples are shown in Figure 9.

Since six species of flying insects should be classified, several SVMs need to be constructed to deal with this multi-group classification problem. The module of 1-v-R (one-versus-Rest) SVMs is employed and six classifiers are constructed in this work. For the training of each SVM classifier, the number of positive samples is around 816 after data augmentation, and the roughly equal number of negative samples are randomly selected from remaining samples. For the testing process, six SVM classifiers are applied for classifying the corresponding species of flying insects. In this way, the system is easily extended if other species of flying insects need to be classified. To avoid over-learning and under-learning, 5-fold cross-validation is employed in our experiments.

## 5. Results and Discussion

In order to test the performance of the proposed system, a strawberry greenhouse and flower planting base were selected as the test scenarios. The detection and coarse counting performance of flying insects based on YOLO method is shown and compared with traditional connected components labeling method. Then, the classification and fine counting performance based on SVM is explained. Next, the architecture of detection with YOLO and classification with SVM is compared with that of YOLO. The simulation platform of connected component labeling counting process and SVM are MATLAB 2014a based on Windows 7, 4G memory and Intel-2467M 1.6GHz and that of YOLO is Darknet depth learning framework based on ubuntu16.04, 8G memory, Intel E5-1620v3 3.5GHz and a GeForce GTX TITAN X graphics card. Finally, the proposed system is implemented on Raspberry PI, and the performance is also tested.

### 5.1. Detection with YOLO and Classification with SVM

#### 5.1.1. Detection and Coarse Counting with YOLO

**Figure 10:**
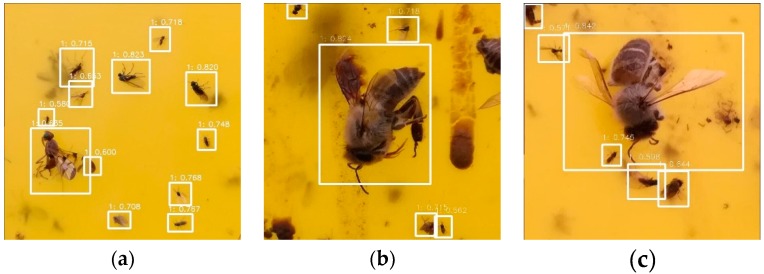
Detection instance of flying insects based on YOLO method. (**a**) Instance 1; (**b**) Instance 2; (**c**) Instance 3.

YOLO deep learning method is adopted to detection and coarse counting of flying insects in our work. Since only flying insect is counted without classification in this process, the parameters are set as *S* = 5, *B* = 2 and *C* = 1. In the testing process, the part where class-specific confidence score is above 0.5 can be labeled. Figure 10 presents the three instances of detection results of flying insects. The counting accuracy is shown in Table 1.

**Figure 11:**
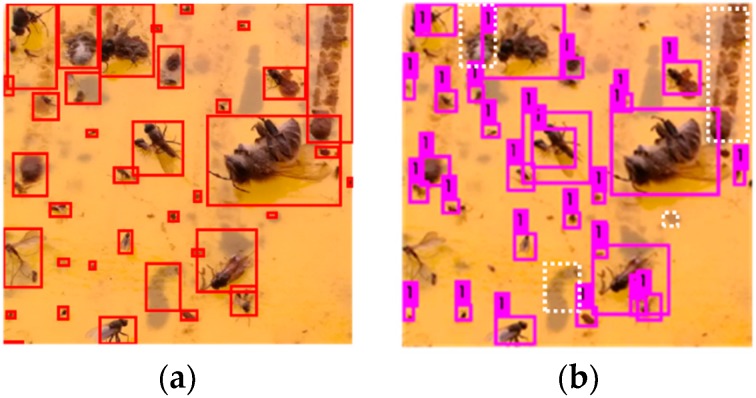
Comparison of detection instances. (**a**) Connected components labeling detection instance; (**b**) YOLO detection instance.

The performance of YOLO is compared with that of traditional connected components labeling method [5]. The instances of detection results of two methods are shown in Figure 11. Figure 11a is the detection instance of connected components labeling method, and Figure 11b is that of YOLO deep learning method. Solid rectangles are used to mark the detected flying insects, but there are some differences between two images. In Figure 11b, white dotted rectangles are used to mark the impurities that YOLO has not detected but connected components labeling method has. More insect excrement, shadow and mud spots are regarded as flying insects in Figure 11a, in contrast, YOLO can distinguish between the impurities and flying insects better.

The counting accuracy of connected component labeling and YOLO is shown in Table 1, which is defined as the ratio of correctly detected number to the totally detected number of flying insects.

The results indicate that the counting accuracy of connected component labeling method is 87.63% and that of YOLO is 93.71%, and it is found that connected component labeling method is easier to be influenced by excrement, mud and other impurities than YOLO, which can also be concluded from Figure 11. Therefore, YOLO depth learning network has higher detection accuracy and stronger anti-interference ability.

#### 5.1.2. Classification and Fine Counting Results of SVM

Considering the impact of kernel function, we evaluate SVM classification performance under different kernel functions at first, and flying insects’ recognition rate based on four kernel functions is compared in Table 2. The kernel function is selected according to recognition performance. The parameters of the kernel function are determined through 5-fold cross-validation and grid search method. From the data in Table 2, it can be seen that the four kernel functions can get good classification effects when the optimal parameters are selected. A radial basis function can get the highest classification accuracy. What’s more, there is little difference among their operation time. Therefore, radial basis function is selected as the kernel function of our SVM classifier.

**Figure 12:**
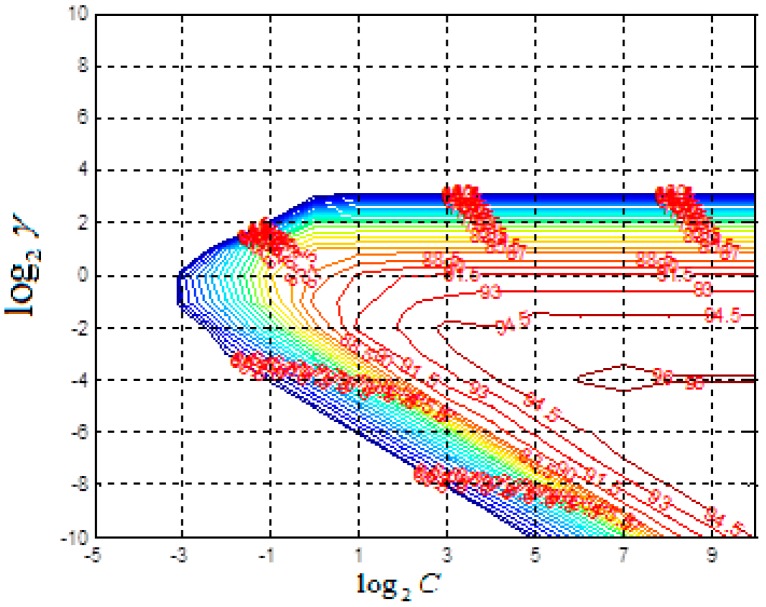
Contour map of grid optimization.

Model parameters of radial basis function mainly include kernel parameter *γ* and penalty *C*. As *γ* becomes bigger, the classification model becomes more complicated, and if *C* is close to infinity, the classifier becomes more accurate, but generalization ability will be affected. Parameters *γ* and *C* are selected and optimized by grid research method. The main steps of parameter optimization are as follows. Firstly, The range of penalty *C* is determined as $\left[2^{-5},2^{10}\right]$ and the range of kernel parameter *γ* is determined as $\left[2^{-10},2^{10}\right]$. Then, the values of *C* and *γ* are selected which is integer power of 2. Next, based on 5-fold cross-validation, the identification accuracy of parameter pairs (*C_i_*, *γ_i_*) is shown in Figure 12. The color of lines can reflect identification accuracy with the blue lines showing low accuracy and red lines showing high accuracy.

Through comparison of classification results, the parameter pair with the highest classification accuracy rate is selected. If there are more than one parameter pair with the highest classification accuracy, the one with the smallest *C* is selected as the optimal parameter pair. If there are more than one group of *γ* with the smallest parameter *C*, the first searched (*C_i_*, *γ_i_*) s selected as the optimal parameter. Therefore, the ultimate selected parameters are C=128, γ=0.0625.

The choice of feature is another factor affecting the performance of SVM classifier apart from kernel function. In this process, the problems of computation amount, computing speed and data redundancy should be considered. The testing set is 2100 samples. And the recognition ability based on color feature, texture feature, shape feature, HOG feature and their combination are tested respectively. It can be seen from Table 3. Classification accuracy is the ratio of correctly classified number to totally classified number of specific species of flying insects.

- If the flying insects are recognized only by their color, texture or shape feature, the recognition rate is limited. For example, bee cannot be recognized well only by color feature and fruit fly cannot be recognized well only by texture feature. Therefore, individual feature cannot fully describe the differences between flying insects;
- The combination of local features and global features can improve the recognition accuracy of flying insects, but recognition accuracy has not been significantly improved. At the same time, the computational amount can increase because more features are considered;
- Based on global features, the recognition accuracy of bees, flies, mosquitoes, moths, chafers and fruit flies are 97.46%, 91.83%, 94.44%, 98.68%, 90.00% and 98.68%, respectively, and the average recognition rate is 95.18%. Therefore, the combination of the three features can improve the classification accuracy, so we choose global features as the classification base of SVM.

### 5.2. Detection and Classification with YOLO 

YOLO can simultaneously predict the bounding boxes and class probabilities for these boxes directly from the entire image, therefore we study the classification performance of YOLO. To achieve promising performance, YOLO needs massive samples to training. However, transfer learning could decrease the requirement of samples [35]. The weights obtained from the training process of natural image in ImageNet are used as the network parameters of the feature extraction layers, and then the flying insect images are only used to train the prediction layer in the YOLO network. To compare with the architecture of detection with YOLO and classification with SVM, we studied the method of transfer learning on YOLO.

In this classification and fine counting process, six species of flying insects need to be classified, considering that other species of flying insects may also exist in the image, so the parameters are set as *S* = 5, *B* = 2 and *C* = 7. The LabelImg tool is used to label the samples. The labels 1–7 are set to represent six species of flying insects and other species, respectively. Three hundred labeled images are used for training. Each image contains several species of insects, and the total number of insect samples is roughly equal with the dataset used on SVM. And the number of samples of 6 species of flying insects is shown in Table 4. In the 300 labeled images, 200 images are used for training and cross-validation, and 100 images are used to test the performance. Due to the change in the number of classes, the number of filters in the last convolutional layer is calculated by (3):

(3) filter=anchor×(class+coor+conf)

where, *anchor* = 5 is the default number of anchor box, *class* = 7 is the number of classes, *coor* = 4 is the number of coordinates, *conf* = 1 is the classification score. Therefore, the number of filters in the last convolutional layer is set to 55.

For the training parameters of the model, the network weights trained on the ImageNet dataset are used as initial parameters, and the learning rate of the feature extraction layer is set to 0. Only the parameters of the prediction layer are optimized using the SGD algorithm. Batch size is set to 8, momentum and weight decay are set to 0.9 and 0.000001, respectively. The initial learning rate is set to 0.0001 and it goes down to 0.0000001 after 5000 iterations.

**Figure 13:**
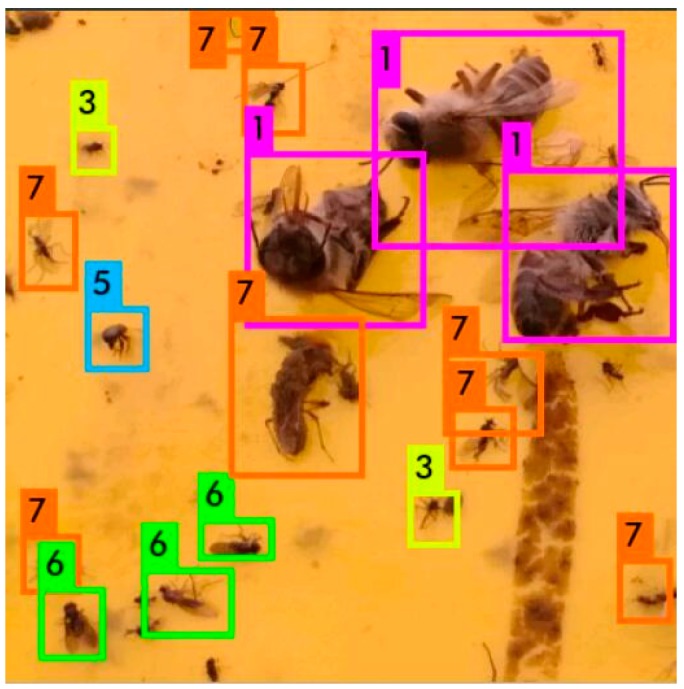
Classification instance of YOLO. The labels 1–7 represent six species of flying insects and other species of flying insects, respectively.

The classification result, the number of samples and classification accuracy of YOLO network is shown in Figure 13 and Table 4. In Table 4, the performance of detection with YOLO and classification with SVM is also presented based on the same 100 testing images.

Since the parameters of feature extraction layers are selected based on the training process of natural image in ImageNet, only the parameters of the prediction layer are optimized based on the dataset of flying insect, hence, the saving training time for the YOLO network. However, the average classification accuracy of this method is low and unacceptable. Compared with detection and coarse counting of only flying insects, category number is larger and tasks are more complicated in classification process. Since the number of samples is only 200 labeled images, the generalization ability is not enough. As a result, the classification accuracy should be improved by increasing the number of labeled samples for YOLO. However, the large number of labeled samples means heavy workload of labeling manually. On the contrary, if regarding all species of flying insects as one class, there is enough sample of flying insects to detection. Combined with a SVM classifier, the performance is better, and it is easy to add or change identified categories of flying insects in the system without retraining the whole system. Therefore, the architecture of detection with YOLO and classification with SVM is selected in our system.

### 5.3. System Testing

As mentioned above, we design the counting and recognition system using YOLO deep learning network to do detection and coarse counting of flying insects and using SVM to do specific classification and fine counting of six species of flying insects. Both processes have obtained good performance as shown in Section 5.1 and Section 5.2. Then, the proposed system is implemented on Raspberry PI. In this section, we test the system based on the images collected from strawberry greenhouse and flower planting base.

**Figure 14:**
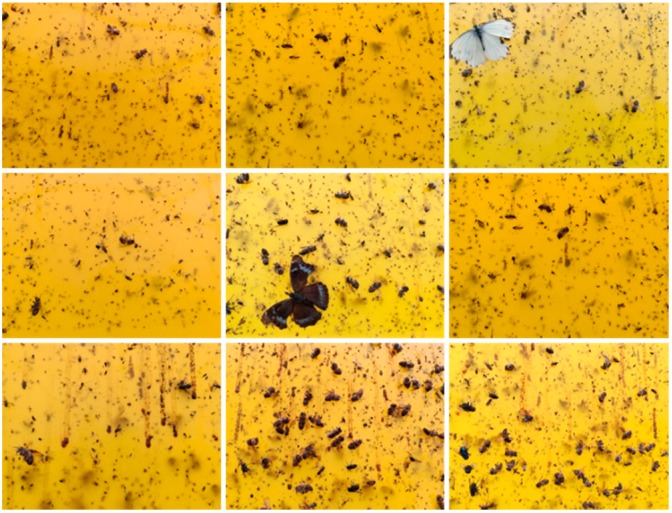
Nine test instances.

The camera captures the images of 3280 × 2464, from the center of which we can extract 896 × 896 images. Then the 896 × 896 image is separated to four equal parts with the resolution of 448 × 448. These 448 × 448 images can be the input of this system. The number of test images is 50. Since the flying insects are rather densely distributed in the image, the total number of flying insects is pretty huge, and we can use statistic results to represent real probabilities. Nine of the 50 images collected in different time and condition are shown in Figure 14.

The average classification and fine counting performance are shown in Table 5. Expert recognition results of flying insects is regarded as the standard. The performance of the system can be evaluated from the aspects of accuracy, recall rate and miss rate. Since the number of impurities is not counted in this system, the false positive rate is not considered. The accuracy is the ratio of the number of correctly detected objects to the total detected number. The recall rate is the ratio of the number of correctly detected objects to the total number of objects contained in the image. Miss rate is the number of objects which are not detected to the total number of objects contained in the image.

The test of 896 × 896 image takes about 5 min on the Raspberry PI at a certain interval which can be adjusted according to requirement to estimate the population dynamics of flying insects. When the flying insect has good gesture and the distribution of them are dispersive, the accuracy is relatively high. If the number of flying insects is large or they are connected with each other, miss rate is relatively high. So the error comes from following aspects. Firstly, the connection phenomenon of flying insects has some influence on the image detection. Secondly, flying insect excrement and other impurities also affect the results. Thirdly, the number of training dataset is limited. To sum up, the accuracy of counting and classification is above 90%, and miss rate is under 7.5%. The proposed system can provide efficient and accurate counting and recognition data which is evaluated by agricultural experts. Consequently, the designed system can be used for intelligent agriculture applications.

## 6. Conclusions 

This study developed a new vision-based statistical and recognition system for flying insects. In this system, we adopt a YOLO deep learning network to detect and coarsely count flying insects and employ SVM to classify the flying insects, moreover, the system is implemented on Raspberry PI. To assess the performance, six species of insects are used to verify the effectiveness of the system. Experimental results show that the average counting accuracy is 92.50%, average classifying accuracy is 90.18% and a cycle of detection and recognition takes about 5 min on a Raspberry PI system. Therefore, this system can reach a relatively high detection rate and efficiency. The main contributions in the paper are summarized as follows:

1. A universal architecture which can detect and identify flying insects was designed, and it is easy to add or change the identified categories of flying insects. The system obtains both the number and species of pests at a certain time interval and sends it to an agricultural service platform, where the population dynamics of pests can be predicted and the suitable pest control strategies can be designed.
2. Deep learning requires a lot of training samples, but it is difficult to get enough samples of some specific insects in a specific season. However, there are enough samples for detection if we take flying insects as one class, therefore, we only use deep learning to detect and coarse counting, and the bounding box of detecting results are provided to SVM to do identification, so as to alleviate the problem of insufficient samples.
3. The proposed system was implemented on a Raspberry PI system. This edge computing design can not only relieve the computing pressure on the server, but also avoid the burden of transmitting high resolution insect images.

In conclusion, the traditional pest detection system based on image processing mainly detects the occurrence of pests and the degree of disease, however, our system can monitor the spread of the insects in real time. Combining the classification and fine counting information of pests with the meteorological information, geographical information and modern technology, an integrated service platform can be formed, which can forecast the occurrence probability and the development trends of pests to provide suitable prevention and control measures to agricultural workers. This kind of integrated service platform is significant and necessary in the field of agriculture.

## Figures and Tables

**Table 1:** Counting accuracy comparison.

Method	Counting Accuracy
connected component labeling	87.63%
YOLO	93.71%

**Table 2:** Performance based on different kernel functions.

Kernel Function	Linear kernel Function	Polynomial Kernel Function	Radial Basis Function	Sigmoid Kernel Function
Support vector number	190	200	165	290
Recognition rate	90.37%	90.37%	92.10%	88.77%
Operation Time	44.6 ms	50.8 ms	52.9 ms	52.7 ms

**Table 3:** Classification accuracy of SVM (Unit: %).

Feature	Bee	Fly	Mosquito	Moth	Chafer	Fruit Fly	Average Accuracy
Shape feature	91.42	97.67	98.43	75.92	60	97.05	86.75
Texture feature	78.57	88.37	96.87	79.62	76	77.94	82.90
Color feature	41.42	72.09	78.12	85.18	68	86.76	71.93
HOG feature	97.46	91.83	91.66	81.66	84.84	97.36	90.80
Global feature	97.46	91.83	94.44	98.68	90	98.68	95.18
Global and local feature	97.46	93.87	97.22	88.33	93.93	98.68	94.92

**Table 4:** Classification performance of YOLO.

Flying Insects	Sample Number	YOLO Only, Classification Accuracy (%)	YOLO + SVM, Classification Accuracy (%)
Bee	562	55.09	91.89
Fly	639	65.53	88.65
Mosquito	591	60.48	90.23
Moth	607	62.11	92.31
Chafer	580	57.31	87.69
Fruit fly	614	63.97	91.25
Average	599	60.75	90.34

**Table 5:** Classification and fine counting performance.

Counting Accuracy	Counting Recall Rate	Counting Miss Rate	Classification Accuracy	Classification Recall Rate	Classification Miss Rate	Test Time
92.50%	93.99%	6.01%	90.18%	92.52%	7.48%	5 mins
